# *Lactobacillus paracasei* Relieves Constipation by Acting on the Acetic Acid-5-HT-Intestinal Motility Pathway

**DOI:** 10.3390/foods12224176

**Published:** 2023-11-20

**Authors:** Linlin Wang, Shurong Yang, Chunxia Mei, Nan Tang, Jialiang Wang, Qiangqing Yu, Gang Wang, Gaojue Wu, Jianxin Zhao, Wei Chen

**Affiliations:** 1 State Key Laboratory of Food Science and Resources, Jiangnan University, Wuxi 214122, China; wanglinlin@jiangnan.edu.cn (L.W.); boyce_yang@126.com (S.Y.); m18862246062@outlook.com (C.M.); 18356788528@163.com (N.T.); 6200112157@stu.jiangnan.edu.cn (J.W.); 6200113228@stu.jiangnan.edu.cn (Q.Y.); wanggang@jiangnan.edu.cn (G.W.); zhaojianxin@jiangnan.edu.cn (J.Z.); weichen@jiangnan.edu.cn (W.C.); 2 School of Food Science and Technology, Jiangnan University, Wuxi 214122, China; 3 Department of Gastroenterology, Jiangnan University Medical Center, Wuxi 214002, China; 4 National Engineering Research Center for Functional Food, Jiangnan University, Wuxi 214122, China

**Keywords:** lactobacilli, constipation, intestinal microbiota, acetic acid, 5-HT, gastrointestinal regulatory peptide, inflammatory factors

## Abstract

Constipation is a major health concern worldwide and requires effective and safe treatment options. In this study, we selected ten strains of two species of lactobacilli to identify whether they were effective against constipation induced by loperamide administration in BALB/c mice. Monitoring of constipation-related indicators indicated that *Lactobacillus paracasei* (*L. paracasei*) mainly acted on the whole intestinal peristalsis to relieve constipation. Furthermore, through the detection of biological, chemical, mechanical, and immune barriers in mice, it was discovered that *L. paracasei* changed the relative abundance of bacteria related to the levels of acetic acid and 5-hydroxytryptamine (5-HT) (such as by increasing the relative abundance of *Odoribacter* and *Clostridium*, and reducing the relative abundance of *Mucispirillum*, *Ruminococcus*, *Coprobacillus*, and *Dorea*), increased the concentration of acetic acid in the intestine, which stimulated enterochromaffin cells, promoted 5-HT synthesis in the colon, enhanced intestinal motility, and relieved constipation. In conclusion, this study provides a theoretical foundation for the development of personalized products for the treatment of constipation.

## 1. Introduction

Constipation is a complex syndrome of gastrointestinal dysfunction. The main symptoms of constipation include decreased spontaneous defecation frequency, difficulty in defecation, dry stool, and low stool volume. Currently, the incidence of constipation in children worldwide is 0.5–29.6% [1], while the incidence of constipation in adults is 1–2.6%, and the incidence in the aged is 4.4–44.5% [2]. The causes of constipation are complicated and varied, and the underlying mechanisms are still not clear, which makes it difficult to completely cure constipation in the clinic.

With the advancement of sequencing technology, the relationship between intestinal microbiota and constipation has gradually been revealed. Compared with wild-type mice, the intestinal motility of germ-free (GF) mice is significantly slower [3]. The feces of specific pathogen-free (SPF) mice were transplanted into GF mice by fecal microbiota transplantation (FMT), the intestinal transmission capacity was thus significantly improved [4], indicating that the intestinal microbiota had a very close relationship with intestinal peristalsis. Similarly, after transplanting the stool of a patient with constipation into GF mice, the contraction ability of the smooth muscle of the mouse colon was weakened, as was the intestinal transmission ability [5]. The above results indicate a close relationship between abnormal intestinal microbiota and constipation. This also made it possible to use probiotics to intervene in the intestinal microbiota to relieve constipation.

*Lactobacillus reuteri* (*L. reuteri*) and *Lactobacillus paracasei* (*L. paracasei*) are common probiotics. *L. reuteri* is very common in the intestines of mammals and has a positive significance in maintaining the normal physiological functions of mammals [6,7]. Currently, *L. reuteri* DSM17938 is being widely used in functional foods. Patients with constipation in the *L. reuteri* DSM17938 intervention group had a 2.6-fold higher frequency of bowel movements per week than the placebo group, and the level of methane was reduced [8]. In addition, studies have shown that *L. reuteri* DSM17938 can have a significant positive effect on abdominal discomfort, pain, bloating, and incomplete defecation in patients with constipation [9]. *L. paracasei* is a probiotic that is widely present in the human intestines, reproductive tract [10,11], and fermented foods, such as cheese and pickles. It is an important member of the human microecological balance [12,13]. Chen et al. found that after intervention with *L. paracasei* NTU 101, the symptoms of constipation in rats were relieved [14]. Second, if patients with constipation took the combination of *L. paracasei* and artichoke for 15 days, the frequency of abdominal distension and incomplete defecation was significantly reduced [15]. Riezzo et al. also found similar results through a population double-blind experiment, where the experimental results showed that the combination of *L. paracasei* and artichoke showed better effects than using artichoke alone in relieving constipation [16].

Although a great number of studies on the regulation of functional constipation by lactobacilli have been carried out in China and other parts of the world, some lactobacilli with the ability to relieve constipation have also been screened, and the possible mechanism by which they relieve constipation, such as improving the composition of intestinal bacteria, has also been discussed. The phenomenon of microbiota imbalance has improved the intestinal microenvironment, but there is still no consensus on how to modulate the intestinal microbiota and improve the composition of the intestinal microenvironment. This study used loperamide to induce constipation to determine the effect of lactobacilli in relieving constipation. We monitored changes in various parameters such as the intestinal microbiota of mice, constipation-related gastrointestinal regulatory peptides, pathological damage of mouse colon tissue, changes in inflammatory factor levels, and the expression of tight junction proteins in the colon, to understand the mechanism by which lactobacilli relieve constipation. This study provides a theoretical foundation for the development of personalized products for the treatment of constipation.

## 2. Materials and Methods

### 2.1. Chemicals and Reagents

Loperamide was purchased from a drugstore. The reverse transcription kit was purchased from Beijing Kangwei Century Biotechnology Co., Ltd. (Beijing, China). Motilin (MTL); gastrin (Gas), substance P (SP), 5-hydroxytryptamine (5-HT), and acetylcholine (Ach) enzyme-linked immunosorbent assay (ELISA) kits were purchased from Shanghai Enzyme Link Biotechnology Co., Ltd. (Shanghai, China).; the claudin-3 protein polyclonal antibody was purchased from Beijing Biosynthesis Biotechnology Co., Ltd. (Beijing, China).; and the gum arabic, acetic, propionic, and butyric acids were purchased from Aladdin (Shanghai, China). The activated carbon solution was prepared according to our previous method and stored at 4 °C until further use [17].

### 2.2. Bacteria Preparation

The lactobacilli strains used in this study were stored in the Culture Collection of Food Microbiology at Jiangnan University and are listed in Table 1. The preparation of all strains used in the animal experiments in this article was the same as our previous methods [18].

### 2.3. Animals and Experimental Design

**Figure 1:**
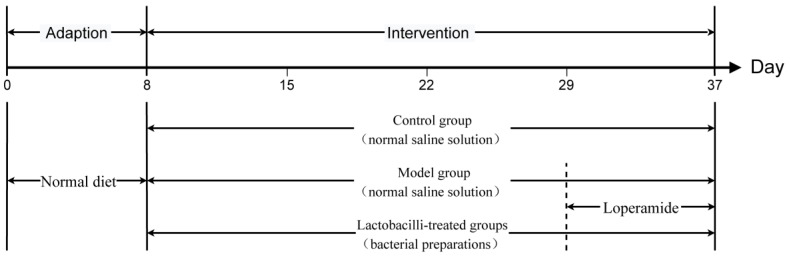
The procedure of animal experiment.

Seventy-two six-week-old BALB/c male mice (Slac Laboratory Animal Co. Ltd., Shanghai, China) were maintained under SPF conditions at the Animal Center of Jiangnan University and acclimated for seven days before the experiments. All mice were randomly allocated to 12 groups (n = 6 each). The 12 groups included a control group, a constipation group, and ten groups treated with different lactobacilli strains, namely, LR1, LR2, LR3, LR4, LR5, LPC1, LPC2, LPC3, LPC4, and LPC5. From days 8 to 29, the mice in the control and model groups were intra-gastrically administered 0.2 mL of saline daily, and the mice in the lactobacilli-treated groups were administered 0.2 mL of bacterial preparations (5 × 10^9^ cfu/mL in saline) daily. All experimental mice were intra-gastrically administered 0.2 mL loperamide, and the control mice were treated with 0.2 mL of saline from days 29 to 37. One hour later, the control and model groups were intra-gastrically administered 0.2 mL of saline, and mice in the lactobacilli-treated groups were administered 0.2 mL of bacterial preparations (5 × 10^9^ cfu/mL in saline). The experimental procedure is shown in Figure 1. All animal experiments were approved by the Ethics Committee of the Experimental Animals of Jiangnan University (JN. NO20180115b1920520) and the study was implemented in accordance with the European Union guidelines (2010/63/EU). After the above treatment, the mice were euthanized with ether anesthesia; blood and gastrointestinal tissues were collected for further analysis. All samples were stored at −80 °C for further analysis.

### 2.4. Faecal Water Content

Feces were collected weekly. The feces were weighed before and after freeze-drying to calculate the fecal water content using the following equation:

(1) $$The\;fecal\;water\;content\;\left(\%\right)=\frac{\left(Wet\;weight\;of\;feces\;\left(g\right)-Freeze-drying\;weight\;of\;feces\;(g)\right)}{Wet\;weight\;of\;feces\;(g)}\times 100$$

### 2.5. Time to First Black Defecation

Mice were fasted overnight (approximately 12 h, with water provided) from Day 35 at 6:00 p.m. After 12 h, 0.25 mL of activated carbon meal was administered to each mouse via gavage. The time from completing gavage to detecting first darkened feces was defined as the time to the first black defecation.

### 2.6. Small Intestinal Transit Rate

Mice were intra-gastrically administered an activated carbon meal (0.2 mL) on day 37. Thirty minutes later, mice were anesthetized using ether, then sacrificed and dissected. The intestine was cut from pylorus to ileocecum and gently pulled into a straight line. Its length was the “total length of small intestine”, while the length from pylorus to the front of activated carbon was the “activated carbon propulsion length”. The small intestinal transit rate was calculated using the following equation [17]:

(2) $$Small\;intestinal\;transit\;rate\;\left(\%\right)=\frac{Activated\;carbon\;propulsion\;length\;(cm)}{Total\;length\;of\;small\;intestine\;(cm)}\times 100$$

### 2.7. Measurement of Gastrointestinal Regulatory Peptides

The levels of MTL and GAS in serum were measured using ELISA kits following the manufacturer’s instructions. The levels of 5-HT, Ach, and SP in the colon tissues were measured as previously described [18]. Briefly, 0.02 g of colonic samples were homogenized with precooled 0.18 mL saline and centrifuged at 12,000× *g* for 15 min at 4 °C. The supernatant was collected and used for 5-HT, Ach, and SP measurements using ELISA kits.

### 2.8. Histopathological Examination

The colons of three mice in each experimental group were selected for staining. The method of Fan et al. [19] was used to stain the colonic mucus layer with Alcian blue. For each slice, the CaseViewer 2.2.1 software was used to enlarge the photo by 40×, and 4 different visual fields were randomly selected. For the thickness of the colonic mucus layer, different sites were selected 6 times in each field, and the average value was taken. The claudin-3 protein in colon tissue was stained according to the method described in the immunohistochemistry kit (Sangon Biotech Co., Ltd., Shanghai, China). Finally, a slice scanner was used for observation and photography. Image J 1.8.0 software and the IHC profile plug-in were used to calculate the positive expression area of immunohistochemical sections. Six regions were randomly selected for statistics in each section.

### 2.9. Quantified Short-Chain Fatty Acids (SCFAs) in Feces

Fecal samples were vacuum freeze-dried, and SCFAs were extracted using the method described in our previous study [20]. SCFAs were analyzed using a gas chromatography-mass spectrometry (GC-MS) system, and the chromatographic conditions were set up as previously described [20].

### 2.10. 16S rDNA Sequencing and Bioinformatics Analysis

DNA was extracted from feces using the Feces Fast DNA Spin Kit (MP Biomedical, Irvine, CA, USA) in accordance with our previous method [20] and was used as a template for PCR amplification of the V3–V4 regions of bacterial DNA. The primers 341F/806R were used as forward and reverse primers, respectively. Briefly, the PCR products were purified using Gene Clean Turbo (MP Biomedical, Beijing, China) and quantified using a Quant-iT PicoGreen dsDNA Assay Kit (Life Technologies, Carlsbad, CA, USA), according to the manufacturer’s instructions. The PCR samples were quantified and amended to equimolar solutions, pooled to establish a DNA library, and sequenced on the Illumina MiSeq platform according to the manufacturer’s instructions. The sequenced raw fastQ data were demultiplexed and quality-filtered using the QIIME2 pipeline with DADA2 [21].

The representative sequences were selected for taxonomic categories against the SILVA database with a 97% threshold, and α-diversity was evaluated using the Chao-1, Shannon, and Simpson indices. β-diversity was assessed from the Bray–Curtis distances and visualized with Principal Coordinates Analysis (PCoA), and the differences were assessed by ANOSIM using the vegan package (R, version 4.0.5). Spearman’s correlation analyses were performed to evaluate the potentially relevant associations between the gut microbiota and constipation-related indicators. The correlation heatmap was plotted using the heatmap package (R, version 4.0.5).

### 2.11. Statistical Analysis

Results are presented as the mean ± standard deviation (SD). Differences were evaluated by analysis of variance (ANOVA) with Dunnett’s post-hoc test (comparison of three or more groups). All regular plots were displayed using the ggplot2 package (R version 4.0.5). The symbol # indicates a significant difference between the model group and the control group (*p* < 0.05), ## indicates *p* < 0.005, ### indicates *p* < 0.001, #### indicates *p* < 0.0005, while * indicates a significant difference compared with the model group (*p* < 0.05), ** indicates *p* < 0.005, *** indicates *p* < 0.001, and **** indicates *p* < 0.0005 (the significance was compared with the model group).

## 3. Results

### 3.1. L. paracasei Relieved Constipation

**Figure 2:**
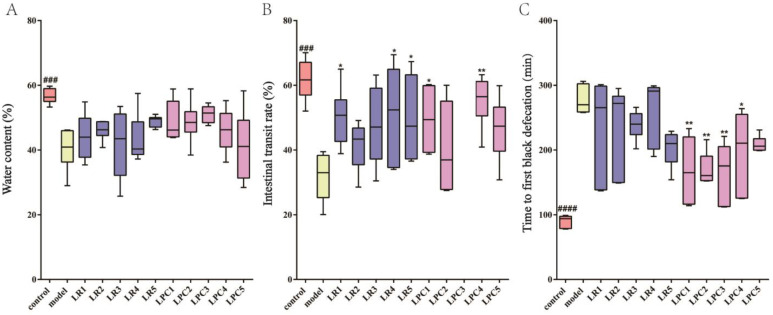
Constipation-related detection indicators. (**A**) the water content of feces; (**B**) intestinal transit rate; (**C**) time to first black defecation. ### *p* < 0.001, #### *p* < 0.0005 (model vs. control); * *p* < 0.05, ** *p* < 0.005 (compared with model group).

As shown in Figure 2, after treatment with loperamide, the fecal water content and small intestinal transit rate in the model group were significantly lower than those in the control group, and the time to the first black defecation was significantly greater than that in the control group, suggesting that the constipation model was successful. Compared with the model group, LR1, LR4, LR5, and LPC1, LPC4 significantly increased the small intestine transit rate (*p* < 0.05). Almost all *L. paracasei* groups significantly decreased the time to the first black defecation (*p* < 0.05), while *L. reuteri* had no effect on the time to the first black defecation. Both *L. reuteri* and *L. paracasei* increased the water content of feces in constipated mice to varying degrees, but there was no significant difference from the model group. In conclusion, *L. reuteri* was ineffective against constipation, whereas *L. paracasei* relieved constipation mainly by modulating the whole intestinal peristalsis.

### 3.2. L. paracasei Had No Effect on MTL and Gas

**Figure 3:**
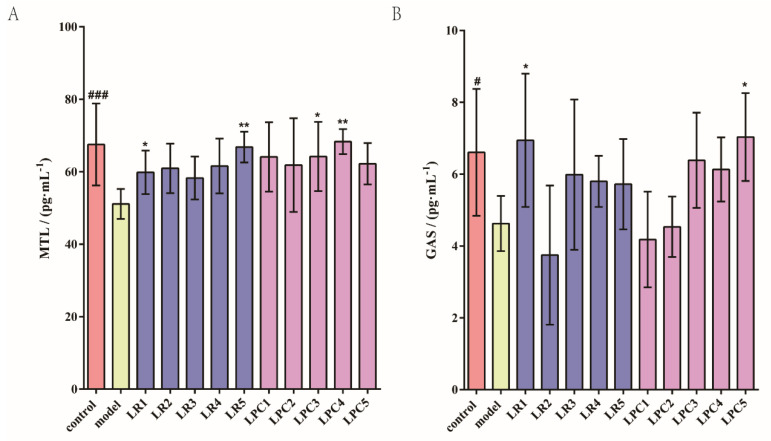
The effect of *L. reuteri* and *L. paracasei* on the level of gastrointestinal regulatory peptides in the serum of mice. (**A**) The level of MTL. (**B**) The level of GAS. # *p* < 0.05, ### *p* < 0.001 (model vs. control); * *p* < 0.05, ** *p* < 0.005 (compared with model group).

As shown in Figure 3, the levels of MTL and GAS in the model group were significantly lower than those in the control group. The levels of MTL in the LR1, LR5, LPC3, and LPC4 groups were significantly higher than that in the model group. The level of GAS in the LR1 and LPC5 groups was significantly higher than that in the model group. Based on these results, and combined with the relief of constipation by *L. paracasei* in Section 3.1, we known that the relieving effect of *L. paracasei* on constipation was not related to the concentration of gastrointestinal regulatory peptides (MTL and GAS) in the intestine.

### 3.3. L. paracasei Up-Regulated the Level of Constipation-Related Gastrointestinal Neurotransmitters in the Colon of Mice

**Figure 4:**
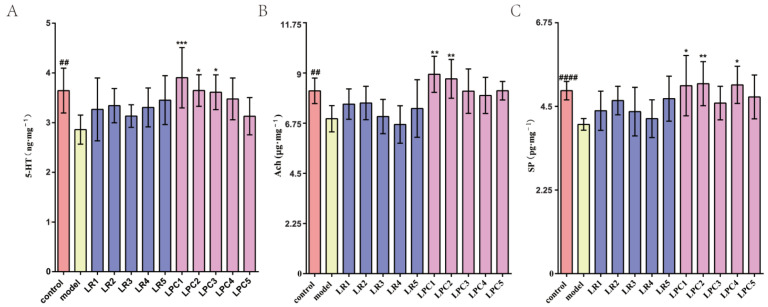
The effect of *L. reuteri* and *L. paracasei* on neurotransmitters in the colon of mice. (**A**) The level of Ach. (**B**) The level of 5-HT. (**C**) The level of SP. ## *p* < 0.005, #### *p* < 0.0005 (model vs. control); * *p* < 0.05, ** *p* < 0.005, *** *p* < 0.001 (compared with model group).

As shown in Figure 4, the levels of Ach, 5-HT, and SP in the colon of the model group were significantly lower than those in the control group. Compared with the model group, only part of the *L. paracasei* intervention groups significantly increased the levels of the above indicators, while the *L. reuteri* intervention group had no effect on the above indicators.

### 3.4. L. paracasei Up-Regulated Percentage of Positive Signal Area of Claudin-3 Protein

**Figure 5:**
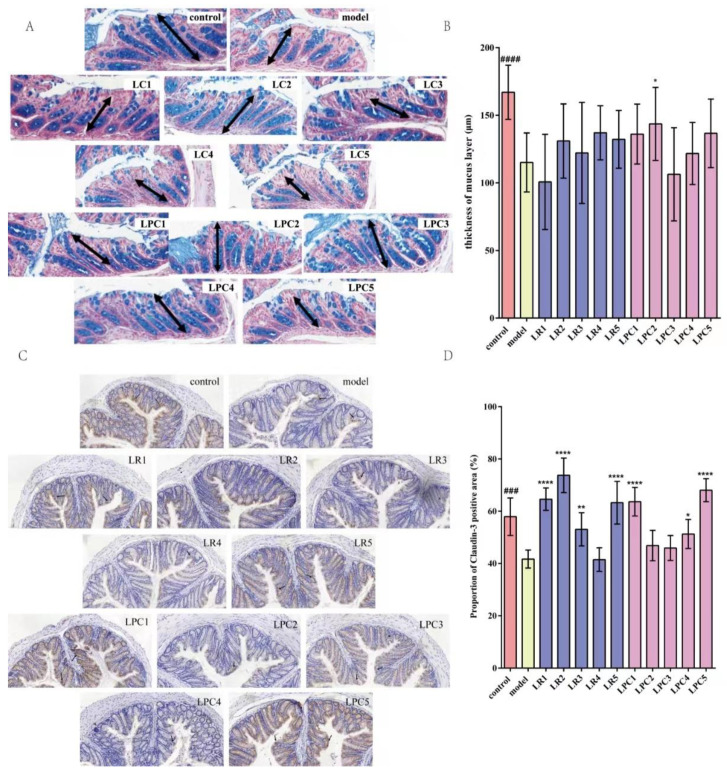
The effect of *L. reuteri* and *L. paracasei* on the intestinal barrier of mice. (**A**) Staining of mucus layer in mouse colon (Arrows indicate the thickness of the mucus layer). (**B**) thickness of mucus layer. (**C**) Immuno-histochemical staining of Claudin-3 protein in mouse colon. (**D**) The percentage of the positive area of claudin-3 in mouse colon. ### *p* < 0.001, #### *p* < 0.0005 (model vs. control); * *p* < 0.05, ** *p* < 0.005,**** *p* < 0.0005 (compared with model group).

As shown in Figure 5, the thickness of the colonic mucus layer and the area of claudin-3 protein in the model group were significantly lower than those in the normal group, suggesting that the intestinal mechanical barrier of the model group was damaged. After treatment with *L. reuteri* and *L. paracasei*, the thickness of the mucus layer of mice treated with LPC2 was significantly higher than that of the model group. The percentage of positive signal area of claudin-3 protein in the colons of mice in the LR1, LR2, LR3, LR5, and LPC1, LPC4, and LPC5 groups was significantly higher than that in the model group. These results suggested that *L. paracasei* relieved constipation by affecting the mechanical barrier of the intestine.

### 3.5. L. paracasei Increased the Level of Acetic Acid in Feces

**Figure 6:**
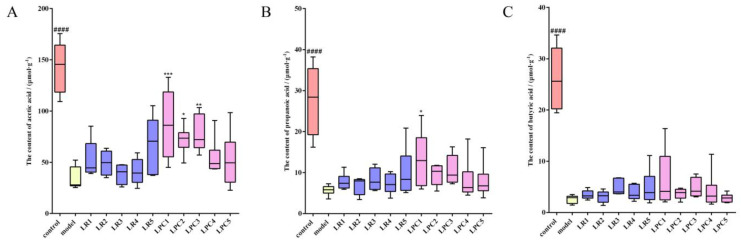
The levels of SCFAs in mouse feces. (**A**) acetic acid; (**B**) propionic acid; (**C**) butyric acid. #### *p* < 0.0005 (model vs. control); * *p* < 0.05, ** *p* < 0.005,*** *p* < 0.001 (compared with model group).

As shown in Figure 6, after loperamide treatment, the levels of acetic, propionic, and butyric acid in the feces of the model group were significantly reduced. Compared with the model group, *L. reuteri* had no effect on the level of SCFAs in the feces of constipated mice, whereas *L. paracasei* mainly affected the level of acetic acid in feces.

### 3.6. L. paracasei Had a Greater Impact on 5-HT, SP and Ach Indices

**Figure 7:**
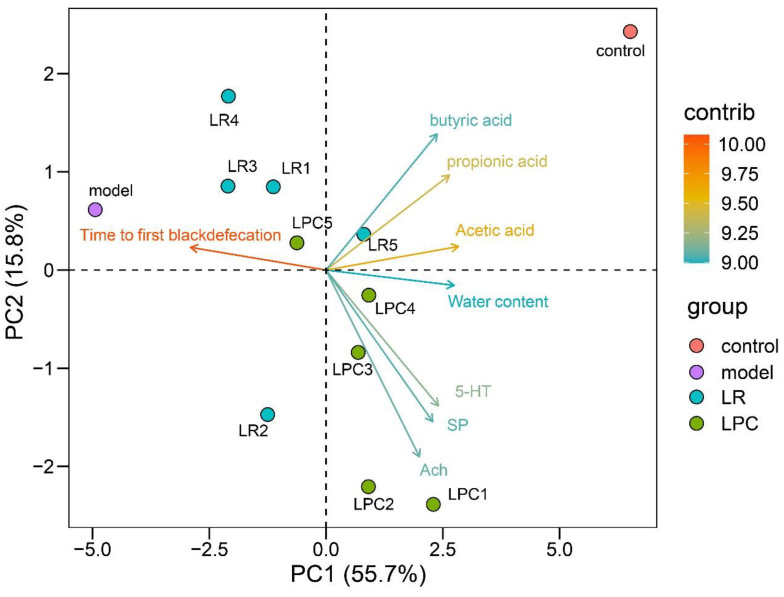
The PCA of physiological indices related to constipation in mice intervened by lactobacilli. Contrib = (var. cos2 × 100)/(total cos2 of the PC component).

The principal component analysis (PCA) of constipation-related indicators is shown in Figure 7. PC is the principal component, in which PC1 and PC2 are the principal components with the first and second contribution degrees, respectively. The PCA diagram shows the contribution of variables with cos2 > 0.8 to PC, where cos2 reflects the representation of each variable in each principal component. The cos2 values of all the principal components of a variable add up to 1. For the principal component, the closer the cos2 of a variable is to 1, the more representative the variable is of the principal component. The closer cos2 is to 0, the less representative the variable is of the principal component. Variables in the same direction and close to each other indicate a positive correlation; variables in the opposite direction indicate a negative correlation. The farther the arrow was from the origin, the higher the representativeness of the PC. As shown in Figure 7, the model and the control group differed greatly in concentrations of acetic, propionic, and butyric acids, while the LR and LPC groups differed greatly in the levels of 5-HT, SP, and Ach.

### 3.7. L. paracasei and L. reuteri Affected the Diversity of the Intestinal Microbiota in Constipated Mice

**Figure 8:**
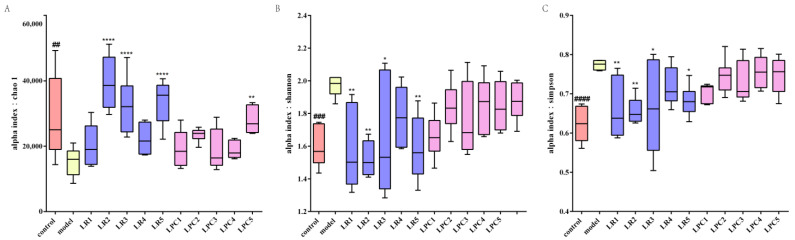
The alpha diversity of intestinal microbiota in mice. (**A**), chao-1; (**B**), Shannon; (**C**), Simpson. ## *p* < 0.005, ### *p* < 0.001, #### *p* < 0.0005 (model vs. control); * *p* < 0.05, ** *p* < 0.005, **** *p* < 0.0005 (compared with model group).

As shown in Figure 8, after treatment with loperamide, the Chao-1 index decreased significantly in the model group, while the Shannon and Simpson indices increased significantly. The LR2, LR3, LR5, and LPC5 groups showed significantly upregulated Chao-1 indices, while the LR1, LR2, LR3, and LR5 groups showed significantly downregulated Shannon and Simpson indices. These results indicated that *L. reuteri* had a greater impact on the α diversity of intestinal microbiota of constipated mice.

**Figure 9:**
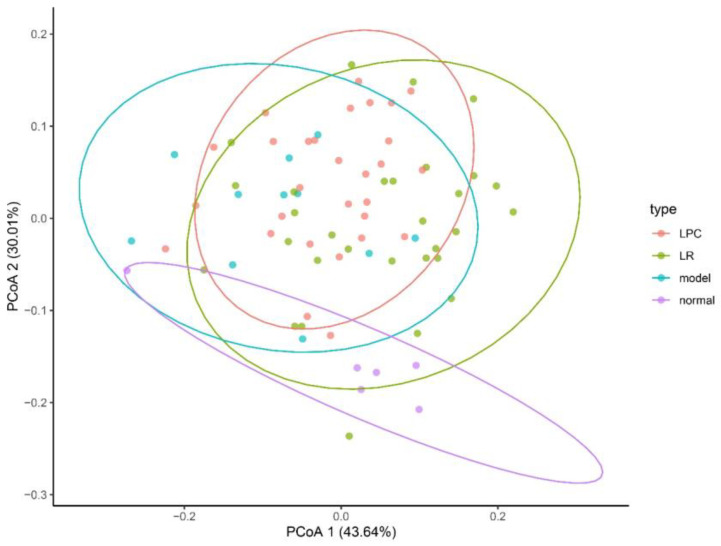
The beta diversity of intestinal microbiota in mice.

As shown in Figure 9, after treatment with loperamide, the β-diversity of the intestinal microbiota was significantly higher than that of the control group. The β-diversity in the lactobacilli group formed a unique intestinal microbiota. However, there was no significant difference between the lactobacilli group and the model group. The changes in intestinal microbiota in different lactobacilli intervention groups at the levels of phylum and genus are shown in Figures S1 and S2 in the Supplementary Materials.

### 3.8. The Mechanism of L. paracasei to Relieve Constipation

**Figure 10:**
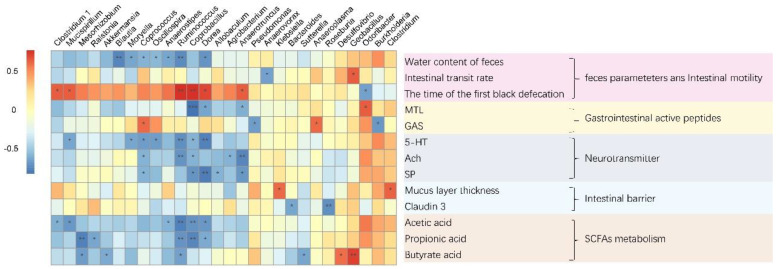
Spearman’s correlation analysis between bacterial genera and constipation-related indicators. The colors ranged from blue (negative correlation) to red (positive correlation), and significant correlations were marked by * *p* < 0.05, ** *p* < 0.01, *** *p* < 0.001.

To explore the association between the gut microbiota and the incidence of constipation, the Spearman’s correlation coefficient was calculated, as shown in Figure 10. *Odoribacter* and *Clostridium* were positively correlated with acetic acid, while *Anaerostipes*, *Ruminococcus*, *Coprobacillus*, *Dorea*, *Mucispirillum*, and *Clostridium* were negatively correlated with acetic acid. The relative abundances of *Odoribacter* and *Clostridium* were positively correlated with 5-HT, whereas *Mucispirillum*, *Moryella*, *Coprococcus*, *Oscillospira*, *Ruminococcus*, *Coprobacillus*, and *Dorea* were negatively correlated with 5-HT. The relative abundance of *Odoribacter* and *Clostridium* was positively correlated with Ach, while *Coprococcus*, *Ruminococcus*, *Coprobacillus*, *Agrobacterium*, and *Anaerotruncus* were negatively correlated with Ach. The relative abundances of *Odoribacter* and *Clostridium* were positively correlated with SP, while those of *Coprococcus*, *Coprobacillus*, *Dorea*, *Allobaculum*, and *Anaerotruncus* were negatively correlated with SP.

Summarizing the correlation results above, it was found that almost all of the bacteria that were related to fecal water content showed a consistent correlation with acetic acid and 5-HT. Combining the relieving effects of *L. paracasei* on constipation, we speculated that *L. paracasei* relieved constipation by changing the composition of the intestinal microbiota and increasing the content of acetic acid. Acetic acid stimulated the release of 5-HT by enterochromaffin cells. The 5-HT further reduced the re-absorption of water in the intestine by accelerating intestinal peristalsis, thereby promoting an increase in water content in feces and, ultimately, alleviating constipation.

## 4. Discussion

The problems caused by constipation to patients are urgent problems to be solved [22]. In this study, we used loperamide to construct a mouse model of constipation. Ten strains of two species of lactobacilli were selected to identify whether they were effective against constipation. The results showed that only *L. paracasei* could relieve constipation, mainly by regulating the peristalsis of the whole intestine. This suggested that not all Lactobacilli can relieve constipation. Some studies had shown that the strain alleviating constipation was related to its adhesion characteristics [23], and some studies had shown that the strain with fast growth rate and short generation time can alleviate constipation [24]. Next, we will study the physiological characteristics of *L. paracasei* and establish the correlation between the physiological characteristics of the strain and relieving constipation, providing a theoretical basis for screening the strain that can alleviate constipation.

Furthermore, the whole intestinal peristalsis was remarkably increased by *L. paracasei*, which may promote the rectification of gastrointestinal regulatory peptide disorders, including Ach, 5-HT, and SP. Ach can excite the smooth muscle of the gastrointestinal tract, increase the contraction range, tension, and peristalsis, and promote the secretion of gastric and intestinal fluids [25]. SP was the first neuropeptide discovered, and it was confirmed as early as the last century that it could significantly increase intestinal motility [26]. The neurotransmitter 5-HT is an important neurotransmitter. More than 90% of 5-HT in the human body is secreted by enterochromaffin cells [27]. Studies have suggested that enterochromaffin cells are important target cells for intestinal microbial metabolites [28]. Research on 5-HT in the regulation of intestinal motility has been conducted for more than 50 years [29]. At present, the 5-HT receptor agonist cisapride has been used in the clinical treatment of constipation. These studies have shown that *L. paracasei* may relieve constipation by increasing the concentrations of Ach, 5-HT, and SP in the colon, thereby decreasing the transit time through the whole intestine.

**Figure 11:**
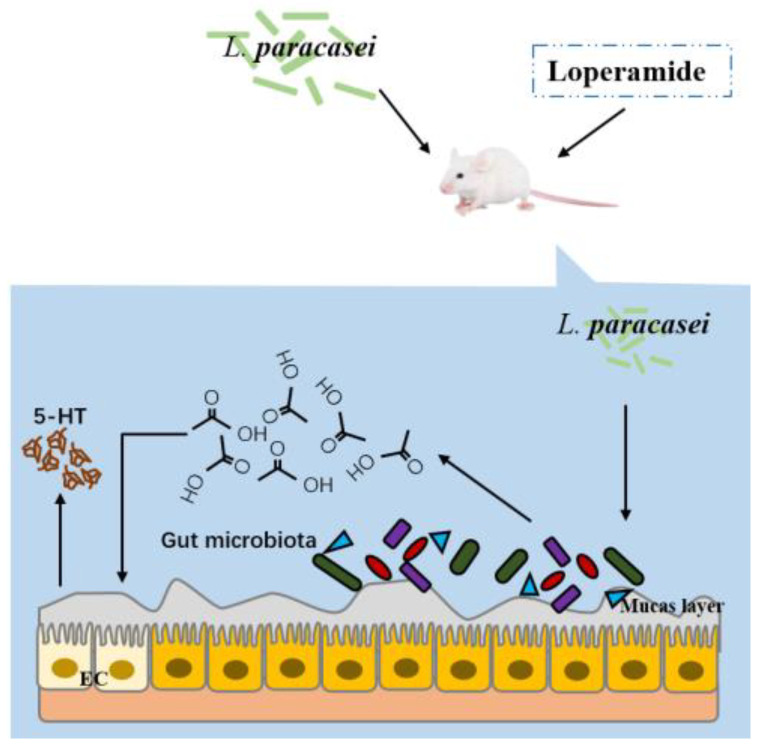
Schematic diagram of the mechanism of Lactobacilli to relieve constipation.

Studies have found that the intestinal transit time of GF mice is slowed down, which is related to the decrease in 5-HT content. When the gut microbiota of wild-type mice was transplanted into GF mice, intestinal peristalsis could be accelerated [30]. This showed that the intestinal microbiota had an impact on intestinal peristalsis. Our research found that constipation was accompanied by intestinal microbiota disorders. It was speculated that intestinal microbiota disorders increased the expression of intestinal serotonin transporter and the re-uptake of excessive 5-HT, reduced the level of 5-HT in intestinal tissues of mice, and promoted the occurrence of constipation [31]. In addition, the disorder of the gut microbiota caused by constipation could ultimately damage the intestinal barrier by reducing the number of intestinal goblet cells (mainly related to the increase in the relative abundance of *Akkermansia*) [31]. Our research found that the thickness of the mucus layer of the constipation group decreased, and the expression of the claudin-3 protein in the colon was significantly lower than that in the normal group, indicating that the intestinal barrier of the constipated mice was damaged. Intervention with *L. paracasei* changed the composition of the intestinal microbiota and increased the acetic acid content in the feces of mice. Our previous studies confirmed that acetic acid relieved constipation [32]. By establishing the correlation between intestinal microbiota and constipation-related measurement indicators, we found that the level of acetic acid was positively correlated with the relative abundances of *Odoribacter* and *Clostridium* and negatively correlated with the relative abundances of *Anaerostipes*, *Ruminococcus*, *Coprobacillus*, *Dorea*, *Mucispirillum*, and *Clostridium*. It was also found that the relative abundances of *Odoribacter* and *Clostridium* were positively correlated with the contents of 5-HT, SP, and Ach in the colon. This showed that the possible mechanisms of *L. paracasei* in relieving constipation are as follows: 1. *L. paracasei* increased the concentration of acetic acid by increasing the abundance of acetic acid-related bacteria, and acetic acid enhanced the water content of feces and colon contractility, thereby reducing the transit time of the whole intestine; 2. Acetic acid directly acted on enterochromaffin cells, promoted colonic 5-HT synthesis, and enhanced intestinal motility. Since the synthesis and metabolism of 5-HT are also directly regulated by the intestinal microbiota, our research also found that *L. paracasei* changed the relative abundance of the bacteria that correlated with the concentration of 5-HT. Based on this, we speculated that *L. paracasei* changed the relative abundance of bacteria related to the levels of acetic acid and 5-HT (for example, by increasing the relative abundances of *Odoribacter* and *Clostridium* and reducing the relative abundances of *Mucispirillum*, *Ruminococcus*, *Coprobacillus*, and *Dorea*) and increased the concentration of acetic acid in the intestine; acetic acid stimulated intestinal enterochromaffin cells, promoted 5-HT synthesis in the colon, enhanced intestinal motility, and relieved constipation (Figure 11).

## 5. Conclusions

In conclusion, we selected 10 strains of two species of lactobacilli to identify whether they were effective at treating constipation induced by loperamide in BALB/c mice. By monitoring constipation-related indicators, our results showed that *L. paracasei* mainly modulated whole intestinal peristalsis to relieve constipation. Furthermore, by detecting biological, chemical, mechanical, and immune barrier-related indicators in mice and establishing correlations between these indicators and apparent indicators of constipation (water content of feces, small intestinal transit rate, and time to the first black defecation), it was discovered that *L. paracasei* changed the relative abundance of bacteria related to the levels of acetic acid and 5-HT (such as increased relative abundance of *Odoribacter* and *Clostridium* and reduced relative abundance of *Mucispirillum*, *Ruminococcus*, *Coprobacillus*, and *Dorea*) and increased the concentration of acetic acid in the intestine. Acetic acid stimulated the release of 5-HT by enterochromaffin cells, while 5-HT reduced the re-absorption of water in the intestine by accelerating intestinal peristalsis, thereby promoting an increase in water content in feces and, ultimately, alleviating constipation.

## Figures and Tables

**Table 1:** The specific information of the experimental strain.

Strains	Abbreviation	Sources
*Lactobacillus reuteri* FYNDL1-3	LR1	faeces
*Lactobacillus reuteri* FCQHC8L6	LR2	faeces
*Lactobacillus reuteri* FHNXY12L1	LR3	faeces
*Lactobacillus reuteri* FZJTZ20M3	LR4	faeces
*Lactobacillus reuteri* FCQNA25M2	LR5	faeces
*Lactobacillus paracasei* FJFZ2L5	LPC1	faeces
*Lactobacillus paracasei* FJSSZ3L1	LPC2	faeces
*Lactobacillus paracasei* FXJWS7M1	LPC3	faeces
*Lactobacillus paracasei* FNMHLBE11L4	LPC4	faeces
*Lactobacillus paracasei* VCQWX3-102L7	LPC5	pickled vegetable

## Data Availability

The datasets generated and analyzed during the current study are available from the corresponding author on reasonable request.

## Supplementary Materials

The following supporting information can be downloaded at: https://www.mdpi.com/article/10.3390/foods12224176/s1, Figure S1: Changes of intestinal microbiota in different treatment groups at phylum level; Figure S2: Changes of intestinal microbiota in different treatment groups at genus level.

## Abbreviation

*L. reuteri, Lactobacillus reuteri*; *L. Paracasei, Lactobacillus. Paracasei*; 5-HT, 5-hydroxytryptamine; GF, Germ-free; SPF, Specific Pathogen Free; FMT, fecal microbiota transplantation; MTL, Motilin; Gas, gastrin; SP, substance P; Ach, acetylcholine; ELISA, Enzyme-linked Immunosorbent Assay; SCFAs, Short-chain Fatty Acids; GC-MS, Gas chromatography-mass spectrometry; PCoA, Principal Coordinates Analysis; SD, Standard Deviation; ANOVA, analysis of variance; PCA, principal component analysis.
