# Supplementary material for: Lactobacillus paracasei Relieves Constipation by Acting on the Acetic Acid-5-HT-Intestinal Motility Pathway
Source: Foods. 2023 Nov 20;12(22):4176. doi: 10.3390/foods12224176 (PMC10670763; doi:10.3390/foods12224176)
Supplement: Supplementary file 1 [file foods-12-04176-s001.zip › foods-2704186-supplementary.pdf]

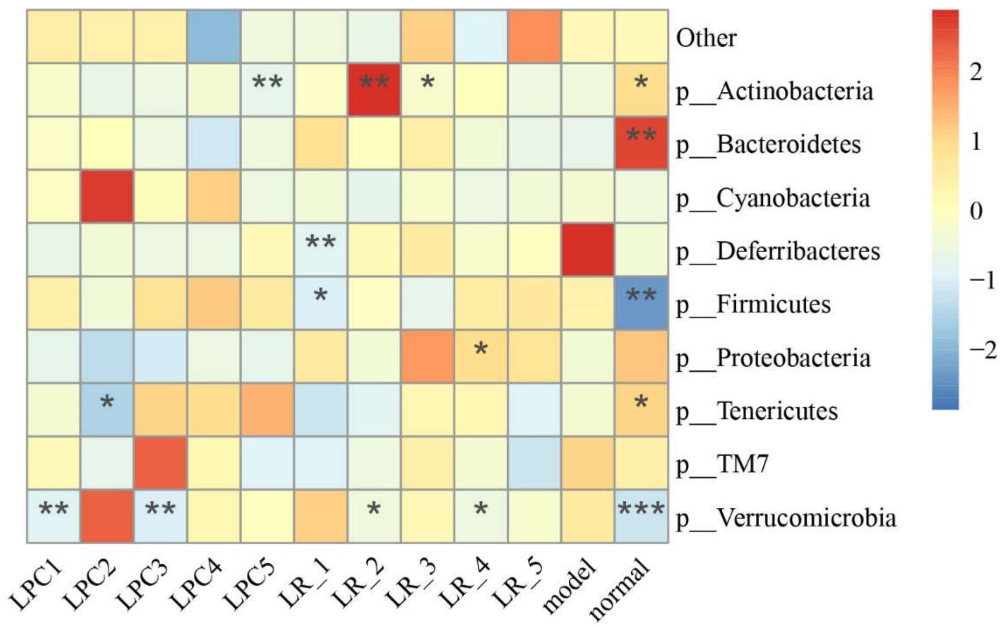

Figure S1. Changes of intestinal microbiota in different treatment groups at phylum level. * Indicates a significant difference compared with the model group. * 0.01<*p*<0.05, ** 0.001<*p*<0.01, *** *p*<0.001.

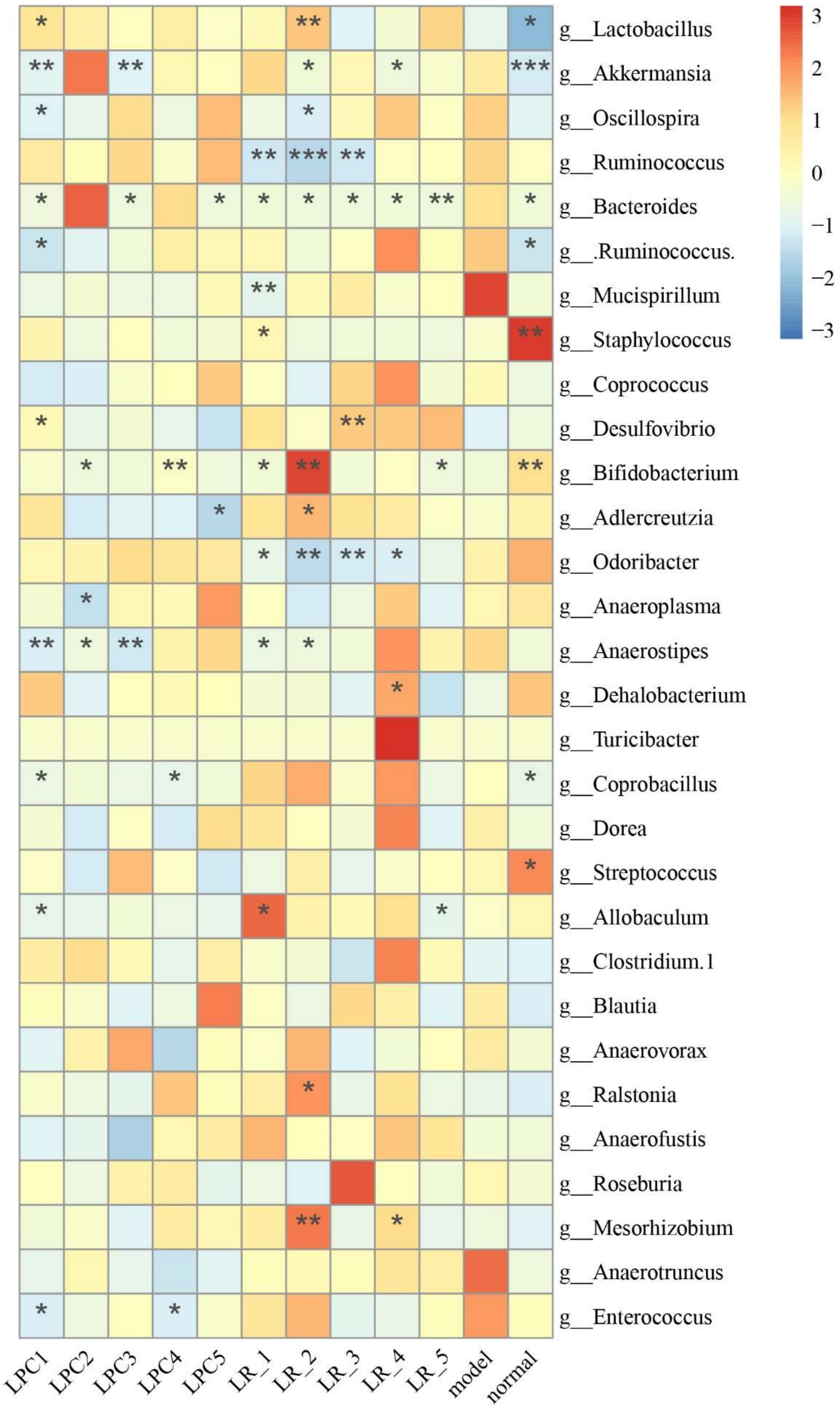

Figure S2. Changes of intestinal microbiota in different treatment groups at genus level. * Indicates a significant difference compared with the model group. * $0.01<p<0.05$, ** $0.001<p<0.01$, *** $p<0.001$.
